# Skin Manifestations of Primary *Vibrio vulnificus* Septicemia

**DOI:** 10.4269/ajtmh.17-0169

**Published:** 2017-07-12

**Authors:** Norman L. Beatty, Jose Marquez, Mayar Al Mohajer

**Affiliations:** 1 University of Arizona College of Medicine, Tucson, Arizona;; 2 Baylor College of Medicine, Houston, Texas

**Figure 1.**
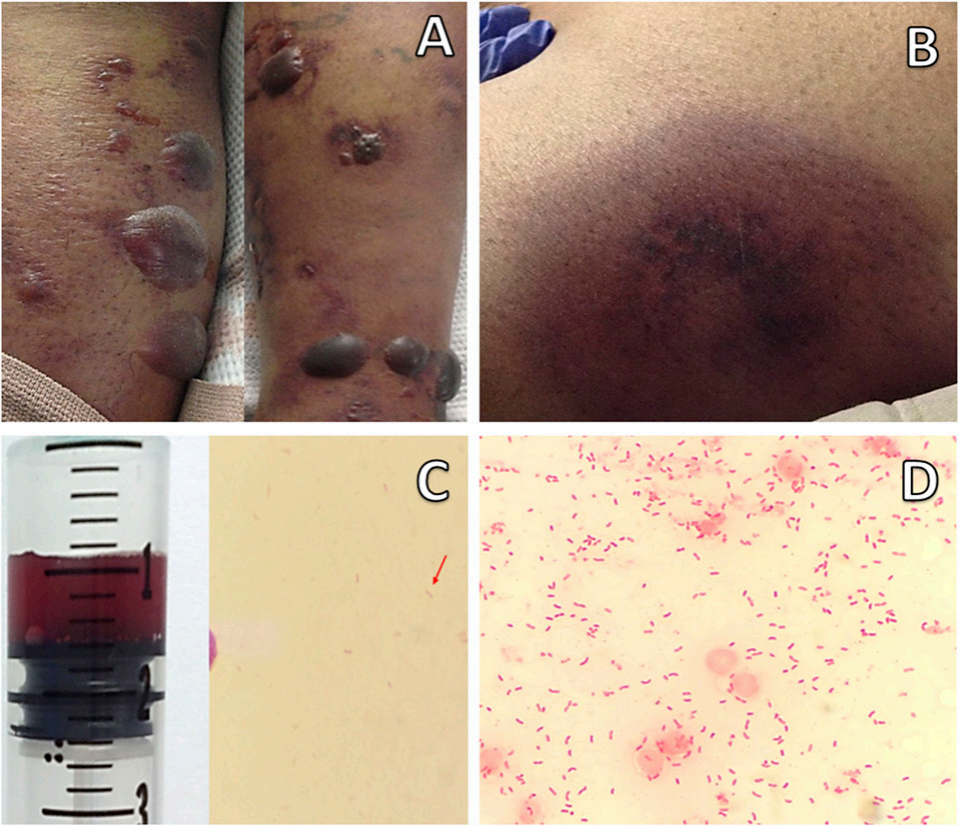
(**A**) Cutaneous bullae with hemorrhagic appearing fluid of bilateral lower extremities. (**B**) Cutaneous ecchymosis appearing prior to developing bullae. (**C**) Bullae aspirate (as seen in syringe) under light microscopy revealing Gram-negative bacilli (red arrow) after Gram staining. (**D**) Innumerable Gram-negative bacilli with some exhibiting curved appearance as seen on Gram stain of blood sample.

A 55-year-old man from the southwest United States (Tucson, AZ) presented with altered sensorium. He has a history of alcohol-induced liver cirrhosis. Family members reported the patient had complained of abdominal discomfort, watery nonbloody diarrhea, malaise, and leg discoloration earlier that day. He had not traveled outside of the United States for more than 20 years. Vitals were significant for hypotension, tachycardia, tachypnea, and hypothermia (35.4°C). Pertinent physical examination findings revealed scleral icterus, abdominal distension, and cutaneous ecchymosis with scattered hemorrhagic appearing bullae located on both lower extremities (Figure 1A and 1B). Laboratory investigations uncovered a white blood cell count 1.2 × 10^9^ cells/µL, platelet count 31 × 10^9^ cells/µL, international normalized ratio 2.1, total bilirubin 5.1 mg/dL, serum bicarbonate 7 mMol/L, blood urea nitrogen 66 mg/dL, creatinine 6.6 mg/dL, and lactic acid 13.0 mMol/L. The Gram stain of the bullae aspirate (Figure 1C) and blood (Figure 1D) revealed Gram-negative bacilli with a curved appearance. Growth from all sampled sources identified the culprit microorganism as *Vibrio vulnificus*. Despite aggressive supportive care in the medical intensive care unit and broad-spectrum antibiotics, the patient unfortunately died from multiorgan failure.

*Vibrio vulnificus* is a Gram-negative halophilic bacilli endemic to coastal regions of warm temperate climates.^1^ Gram staining will often reveal a short, slim, and curved Gram-negative bacillus under light microscopy. *Vibrio vulnificus* is a potentially lethal pathogen and the leading cause of seafood-related death in the United States.^2^ High rates of infection are reported in Taiwan, South Korea, Japan, and Gulf of Mexico of the United States.^1^ Exposure to *V. vulnificus* occurs either through direct contact with a contaminated water source or seafood (handling or ingestion) which can lead to gastroenteritis, skin and soft tissue infection (often necrotizing), or septicemia.^2–4^ Our patient was lacking any contact with a salt water source but did ingest shrimp purchased from a roadside stand 2 days prior to his presentation. Septicemia can be a primary process (foodborne) or secondary to an invasive wound infection.^1–3^ Liver disease is the most notable risk factor for developing primary *V. vulnificus* septicemia with 96% of patients reporting consumption of raw or undercooked oysters within 7 days of illness.^4,5^ The case fatality rate is estimated between 34% and 61% in this population.^2–5^ In primary *V. vulnificus* septicemia, erythematous and bullous skin lesions can appear within the first 24 hours after symptom onset, indicating an early sign of a potentially fatal disease.^2,5^
